# A Review of the Benefits of Nature Experiences: More Than Meets the Eye

**DOI:** 10.3390/ijerph14080864

**Published:** 2017-08-01

**Authors:** Lara S. Franco, Danielle F. Shanahan, Richard A. Fuller

**Affiliations:** 1 School of Biological Sciences, University of Queensland, Brisbane, QLD 4072, Australia; R.fuller@uq.edu.au; 2 Zealandia Sanctuary, 31 Waiapu Road, Karori, Wellington 6012, New Zealand; Danielle.shanahan@visitzealandia.com

**Keywords:** sensory, nature benefits, nature experience, wellbeing, nature therapy

## Abstract

Evidence that experiences of nature can benefit people has accumulated rapidly. Yet perhaps because of the domination of the visual sense in humans, most research has focused on the visual aspects of nature experiences. However, humans are multisensory, and it seems likely that many benefits are delivered through the non-visual senses and these are potentially avenues through which a physiological mechanism could occur. Here we review the evidence around these lesser studied sensory pathways—through sound, smell, taste, touch, and three non-sensory pathways. Natural sounds and smells underpin experiences of nature for many people, and this may well be rooted in evolutionary psychology. Tactile experiences of nature, particularly beyond animal petting, are understudied yet potentially fundamentally important. Tastes of nature, through growing and consuming natural foods, have been linked with a range of health and well-being benefits. Beyond the five senses, evidence is emerging for other non-visual pathways for nature experiences to be effective. These include ingestion or inhalation of phytoncides, negative air ions and microbes. We conclude that (i) these non-visual avenues are potentially important for delivering benefits from nature experiences; (ii) the evidence base is relatively weak and often based on correlational studies; and (iii) deeper exploration of these sensory and non-sensory avenues is needed.

## 1. Introduction

Experiences of nature provide people with multiple benefits to health and well-being, yet the mechanisms by which these benefits are delivered are not well understood [1,2]. Interest in nature as a therapeutic resource has ancient foundations. Hippocrates extolled the necessity of “airs, waters, and places”, for physical and mental well-being [3], and ancient Roman texts suggest that there are health benefits to countryside and greenspaces [4]. Gardens were prescribed for monasteries in the 1200’s “not only for food, but also for recreation in the open air to aid the recovery of the sick and to preserve health and improve those fatigued by their spiritual studies,” according to the Franciscan Minister General, Bonaventura, in 1260 [5]. In 1839, the Annual Report of the British Registrar General opined that, “a park in (the) East End would diminish annual deaths by thousands and add several years to the lives of the entire population” [4]. Overexposure to manmade environments was believed to cause “excessive nervous tension, over-anxiety, hasteful disposition, impatience and irritability” [6]. An early American illness known as neurasthenia with symptoms of depression, anxiety, insomnia, and migraines, was often cured with nature therapy, known as the “west cure”, where men (including prominent figures such as poet Walt Whitman, painter Thomas Eakins, novelist Own Wister, and US President Theodore Roosevelt) were sent west to ranches to work roping horses on the range [7]. The cure they suggested was simple: experiences of pleasant rural scenery. This idea has spawned an enormous volume of research on the visual appeal and restorative potential of natural landscapes [8,9,10]. However, emphasis is repeatedly placed on experiences of pleasant rural *scenery*. Perhaps as a result of vision being the dominant human sense, research has focused heavily on the visual benefits of nature experiences potentially at the expense of understanding the non-visual senses, and other pathways such as airborne volatile chemicals and the ingestion of microbiota. Emerging evidence is pointing to the existence of a broad range of sensory and non-sensory pathways for the benefits of nature experiences. Yet the research base isolating sensory experiences of nature beyond vision is sparse. In this paper, we review the state of this evidence so far, and identify some important gaps in our understanding of how nature experiences benefit human health and well-being.

The multi-sensory aspect of nature experiences is crucial because monotony of stimulation can be a source of stress [11] and multimodal sensory input itself can drive positive mental states such as tranquility [12]. Indeed, it has been shown that stimulating multiple senses at the same time may possibly lead to additive beneficial effects of nature experiences [13]. For example, one study found that while a virtual nature environment was able to reduce stress in participants, these participants also felt negatively towards the virtual environment, and expressed a sense of missing the full sensory experience of real nature [14]. This example highlights the possible shortcomings of assuming visual delivery is the dominant pathway through which nature benefits are delivered.

In this paper, we review the ways in which we experience nature through each of our senses and through several non-sensory pathways. Most of the literature we review focuses on the passive reception of benefits, but it should be recognized that there may be benefits derived from a more active engagement with the environment. More specifically, there may be a difference between passive sensation, and the next step after it of processing to perception.

## 2. Methods

This paper is intended to be a narrative review of disparate literature designed to provide a reference for wider reading rather than to provide a systematic review of the evidence. As such, no systematic search or synthesis has been attempted and instead, a number of search terms were used and anything considered relevant to senses and nature benefits was included. Multiple study designs were included, as well as research on animals in addition to humans. Some search term examples, for sound, included “sound”, “noise”, “nature benefit”, “wellness”, “health”, “wellbeing”, with similar searches for the other senses. When relevant articles were found, a snowballing method was utilized, searching their references for further relevant articles. In some instances where very few results were found, we included preference studies as well as correlational studies where the effects of possible confounding variables could not be assessed.

We define nature in a broad sense as “the phenomena of the physical world collectively, including plants, animals, the landscape, and other features and products of the earth, as opposed to humans or human creations” [15]. We consider nature to include phenomena as varied as landscapes, microorganisms, and pets, and we also include nature simulations. We consider health as “the state of being free from illness or injury”, and as a statement about one’s mental or physical condition [15]. Finally, we define wellbeing as “the state of being comfortable, healthy, or happy” [15], and including self-acceptance, personal growth, purpose in life, environmental mastery, autonomy, and positive relations with others [16]. It further includes the important domains of mental well-being, social well-being, physical well-being, spiritual well-being, activities and functioning, and personal circumstances [17]. Therefore wellbeing is a very broad category encompassing concepts as varied as “freedom from noise” and “memory recall”.

Senses can be thought about in two broad ways: as passive reception and as active searching. Our paper has not differentiated between the two, but focuses more on the passive sense as there seems to be more literature here. It would also be possible to look at perception (that is a further step away from sensing and includes some processing). Much of the literature does not seem to make these distinctions and this may be an area of possible future research.

## 3. Sight

Viewing nature has been repeatedly demonstrated to provide a range of benefits for human health and well-being [18]. Benefits include reduced anxiety [19], reduced stress [20], shorter hospital stays [21], lower heart rate [22], and increased directed attention [23]. The duration of these benefits has not been investigated and is an avenue of possible future research. Hospital patients recovering from surgery showed greater positive affect, received fewer negative notes from nurses (where nurses recorded the patient’s mood and attitude) and spent less time in hospital if their room overlooked trees instead of a brick wall [21]. In another study, pictures of art depicting nature (trees, greenery, flowers, and water) were rated positively by hospital patients, while abstract art increased anxiety, suggesting that nature content, itself, was important [24].

It remains unclear precisely which elements of a view of nature are beneficial, hampering the design of natural therapy interventions or of urban greenspaces themselves. Three quarters of studies only make a coarse division between “urban” and “nature” when studying the beneficial effects of nature views [18], and so we remain unsure which visual elements of landscapes are responsible for the benefits. It may be that only a combination of elements in a coherent scene confers benefits, or it may be that individual elements alone are sufficient. For example, the colours of nature could be important. Blues and greens, which predominate in nature scenes are low-arousal, low-anxiety, and highly preferred colours [25,26,27], while the gray colours of urban scenes seem to result in feelings of aggression [28] and dominance [25].

There are other possible visual cues that could make nature environments restorative: the lack of straight lines, the shape of the vegetation, and visual variety in the scenery. A study by Berman et al. [29] found that naturalness was associated with density of contrast changes, density of straight lines, average color saturation, and average hue diversity. Another study found that curves and hue diversity had effects on preference [30]. Fractals, which are found in many natural images, may also have some role in aesthetic preference [31].

While there is plentiful literature showing the health and well-being benefits of visual nature, it remains uncertain which elements of this visual experience contribute to the benefits. In a review of comparisons between moderately realistic computer animations and the corresponding real environments it was suggested that the former do not generate the same self-reported cognitive and affective responses as the latter [32], implying that while the visual aspect of the environment does drive some benefits, it is far from the only likely mechanism.

## 4. Sound

Based on the number and relevance of literature found, hearing appears to be the second most-studied of the human senses. Hearing is the perception of acoustic waves that provide us with information about the environment. Our auditory systems are evolved to be most sensitive to sounds that are most important to our survival and reproduction [33]. Natural sounds, usually considered the most complex and informational of sound types, can provide information on species, season, and temporality [34], and it is likely that we are attuned to such cues. Indeed a component of attention restoration theory is that restorative environments provide us with information [35], and it thus seems plausible that the rich information content of nature sounds contributes to the restorativeness of natural landscapes. Sounds are also a component of place attachment [36] and are felt as a link to the environment [36,37,38], both of which could be associated with positive feelings about one’s environment [39].

It has repeatedly been shown that the sounds of nature such as wind, water, and animals, are preferred over anthropogenic sounds such as traffic, recreational noise, and industrial noise [40,41,42,43,44,45]. With respect to perceived restorativeness, rural soundscapes and botanical gardens were preferred over urban park soundscapes, which were preferred over urban soundscapes [46]. Preferred environments have been found to be correlated with restorative potential [47,48], so the existence of positive preferences for nature sounds implies, though does not demonstrate, that they might themselves be restorative. Indeed, bird sounds have been found to increase recovery of skin conductance level, a measure of stress [49], and visitors to a local river cited the sound of water as a reason to visit a local river for its relaxing effects [50].

Nature sounds have been used therapeutically to relieve stress [49,51,52], and perceived restoration and attention recovery have shown positive reactions to birdsong [53]. A virtual reality forest including sound was found to improve stress recovery more than the same forest without sound, implying that the sympathetic nervous system shows increased recovery with nature sounds [54]. A zoo exhibit including rainforest sounds was rated as more pleasant than the same exhibit without sounds [55], and natural sounds have also been found to decrease self-reported anxiety and agitation [56].

Images of cities receive higher ratings of perceived pleasure when paired with nature sounds, especially water, while urban sounds were found to decrease the ratings of natural images [42]. Ratings were especially high for nature image and sound combinations, as “apparently sounds provide a specific kind of information over and above the visual which helps enhance and emphasize the different components of the environment” [42]. Sound combined with 3D images increased realism and preference ratings when it was congruent [57]. Appraisals of places depend on the sounds heard there [58,59], highlighting the importance of sound. Sounds have been found to affect the perceived realism of simulated environments [60], as well as their perceived naturalness, solitude, and freedom [45,61,62,63]. At low levels, nature sounds were found to decrease perceived crowding and increase interpersonal encounter tolerance [64], while fountain and bird sounds were found to decrease perceived loudness of traffic and enhance soundscape pleasantness and eventfulness [65]. Indeed, the absence of human-made sounds allowing for the perception of nature sounds is considered to be very pleasurable [66].

### 4.1. When Sound Becomes Noise

Noise pollution has become an increasing public complaint in the last decade [67], and some 80 million Europeans live with unacceptably high urban noise levels [68]. Chronic noise contributes to stress, annoyance, cardiovascular problems, sleep disturbance, and decreased task performance [69,70,71]. It has both psychological and physical effects ranging from elevated blood pressure, poor sustained attention, and memory problems to sleep disturbances, increased risk of myocardial infarction, annoyance, and learned helplessness [72,73,74,75,76,77,78]. These effects can occur below our level of awareness [79].

Since noise negatively affects our health and wellbeing, the respite from it found in nature is potentially an important benefit. Seeking freedom from noise is likely to be a driver for many to seek out natural soundscapes and recreational nature experiences [80]. “Natural quiet”, that is natural sounds without anthropogenic noise, is considered by the US Congress and National Park Service as an important resource to be protected [81,82,83]. Vegetation itself has a significant dampening effect on noise, and this is a well-reviewed ecosystem service [84].

Quiet and natural sounds increase the quality of visitors’ experience in parks [72], and escaping noise to enjoy natural sounds is an important reason for visiting parks [85]. Supporting this, 91% of Americans cite enjoyment of quiet and natural sounds as a reason to visit national parks [86], and 72% believe that a reason to protect national parks is to preserve the natural quiet and nature sounds [87], suggesting they consider them to be of some benefit. Sounds considered most pleasing include water, wind, birdsong, and bird chatter [45], and these sounds were found to be pleasing to campers and mountaineers [88]. Visitors to natural parks are sensitive even to low levels of anthropogenic noise, which significantly detracts from their enjoyment [79].

### 4.2. Warnings in Silence

If quiet is so important, why is silence in an urban environment not a good thing the same way it is in a natural environment? While “peace and quiet”, as described in the previous section, is frequently sought out by humans looking for restoration, auditory deprivation in a laboratory setting increases anxiety [89], and total silence in a natural environment might well have negative connotations. In the earlier example of a virtual reality forest which, with sound was found to increase stress recovery, the same forest without sound was found to induce apprehension and a fear of threat [54]. This may be because silence in nature can indicate predator presence or depauperate biodiversity. Indeed, participants in the Annerstedt et al. [54] study indicated that they felt a predator was about to reveal itself in the silent virtual forest.

It is possible that over the course of our evolution, we came to identify silence with the response of most animals to a predator’s presence. It has been suggested that humans evolved humming as a way to signal safety during quiet times so there would be no silence [90]. Other animals perceive and use silence as a danger warning. Many insects, for example, become silent when they detect a predator [91], as do tungara frogs, *Engystomops pustulosus*, which use the silence of neighbours as an alarm cue to silence their own singing [92]. In fact, animals frequently detect predators “vicariously” through the alarm response of others [93,94,95], remaining in “adaptive silence” [96].

Together this suggests that to animals (including humans), a silent landscape would generally prove disturbing or unnerving [42]. In the urban environment, we are often faced either with situations that have an overabundance of anthrophony (human-generated noise), or an absence of any noise at all (e.g., a deserted alleyway late at night). Both situations are devoid of the reassuring animal noises that can be identified with a safe (i.e., predator-free) and abundant (i.e., prey-full) environment. Therefore, in addition to nature sounds providing the positive benefits described above, a lack of nature sounds could contribute to negative outcomes such as apprehension and anxiety.

## 5. Smell

Smell is one of our weakest senses, yet the world around us emits all kinds of smells. Smells are everywhere, and this is true of both urban and natural environments. The main difference between these two environments is the abundance of anthropogenic smells in urban environments, and the lack of them in natural environments. Nature abounds in smells from flowers, trees, shrubs, grasses, animals, rotting matter, and insects. How these smells affect our health and wellbeing is an interesting, but relatively poorly studied question.

Smell can have profound effects on our mood, behavior, and cognition. Many natural odors are commonly found to be pleasant. For example, thoughts about the odors of flowers, cut grass, and damp earth, might evoke feelings of pleasure. However, there are also odors associated with information, such as the distaste we feel at the odor of rotting meat signaling its unpalatability. Some of the benefits of natural odors are the pleasant affective states they induce, as well as the warnings they have about potential toxicity. The odors of summer air (leaf alcohol) and bees wax have been shown experimentally to be associated with the emotion of happiness in participants [97], and in an experiment in a fragrant garden, “natural odors derived from blooming plants increased calmness, alertness, and mood” [98]. The natural odors of lavender and spiced apple have been shown to alter brain activity, and thus affect cognition as measured by EEG beta and theta waves, which show changes similar to those seen during cognitive tasks involving internal verbalization [99]. Our preferences for odors seem to be associated with the value we place on the objects associated with that odor [100], so smells that remind us of the outdoors can trigger any of the positive feelings we have about nature. Nature smells can thus function as a kind of trigger or symbol for nature in general and might deliver nature benefits by proxy. So while nature smells can have direct nature benefits, they can also have indirect nature benefits.

As in the case of rotten meat and the disgust response to the smell, smells can provide us with useful information about the environment, or specific resources or organisms within it. In a study on humans, it was found that men and women could identify whether individuals were happy or fearful based on odor pads used to collect their sweat [101]. Natural odors from plants and animals such as floral fragrance and musk, are indicative of metabolism and physiological function [102], so smells can tell us about the state of possible food.

One of the ways olfactory molecules can affect us is the link between the sense of smell and the limbic (emotional) system [103,104]. Emotions and odors are connected [105,106,107], and olfactory disorders can manifest as depressive episodes [107]. The olfactory system is also connected to hunger (hypothalamus), and memories (hippocampus) [108,109], and affects mood [110,111,112], cognition [99,113], and behavior [114,115]. This suggests a clear potential avenue through which nature benefits could be received via smell, yet there are very few studies on the role of smell in delivering health and well-being benefits of nature experiences.

Given the paucity of work on the link between smell and benefits from nature experiences, some results from studies not directly focused on natural smells suggest some intriguing possibilities. In a study on attraction and social perception, it was found that perfume (a pleasant scent) had an effect on perceived attractiveness and perception of the user’s traits [116]. It has also been found that odors can cause a change in a person’s liking rating of a face in a photograph [117]. Helping behaviors (such as picking up a dropped pen) increase during exposure to pleasant odors such as cookies or coffee, as does positive mood, which may have a mediating effect [118]. Individuals exposed to a floral and a lemon fragrance performed better on an anagram word task, as well as being more willing to volunteer for a task afterwards [119]. Because these effects also occurred when a small gift was given, it is theorized that these effects, too, are mediated by positive affect.

Exposure to both muguet (a relaxing fragrance) and peppermint (an alerting fragrance) increased signal detection by participants in a 40 min visual sustained attention task, indicating enhanced vigilance performance [120]. In the context of learning, it has been found that subjects exposed to the odors of jasmine or perfume while learning recalled a list of words better when they were exposed to the same scent from when they learned the words the first time, indicating odor as a possible memory eliciter [121].

These results from some artificial and nature-mimicking odors implies that there are well-being benefits from smell, and this area of nature experiences seems likely to repay further investigation.

### Essential Oils

Plant essential oils have long been thought to have physiological and psychological effects according to folk belief [112], and fragrances have been used for their effects on health and mood [122]. Essential oils are typically obtained by distillation from plant material and have the characteristic odor of the original tissue. There is much anecdotal evidence that aroma molecules affect human behavior and physiology, as well as memory activation and mood [123,124], yet experimental evidence is sparse and often equivocal. Essential oils have been shown to decrease depression, anxiety, stress, and blood pressure [125,126,127], and different oils have been found to have different effects [128,129]. It remains unknown whether the effects of documented and/or claimed for essential oils also occur in situ in places where the sources of the essential oils occur in natural environments.

In humans, a review found that there is some support for using sensory interventions, including aromatherapy, in the treatment of the behavioral symptoms of elderly dementia [130], and another review found some evidence that aromatherapy lowered blood pressure in patients with hypertension, but suggested there was a need for more studies with adequate controls [131].

Reviews of the effects of essential oils suggest the evidence so far remains contradictory. For example, Cavanagh and Wilkinson [132] concluded that there is clinical and scientific evidence supporting the use of lavender oil for traditional uses such as calming and anti-depressant effects, despite methodological and oil identification issues. However, another review on aromatherapy concluded that it only had mild anti-anxiety properties, with no other effects supported by clinical trials [133]. A study on aromatherapy use by nurses found that there was not enough empirical evidence (other than for enhancing relaxation) to support its use [134]. However, a review on studies on older patients with dementia found that essential oils did have an effect on cognitive function and independence of daily activities [135]. It has also been found that essential oils have pro-oxidant effects at the cellular level, with anti-mutagenic effects, and thus presumably anti-carcinogenic effects as well [136]. It is quite well established that some essential oils have antimicrobial effects, but this is usually tested with topical application, rather than through the volatile element [137]. It makes sense that essential oils would have biological effects, as they are produced by plants to protect against herbivores and pathogens and to attract pollinators [138], however, evidence seems to be predominantly anecdotal thus far. Further experiments on clinical benefits are needed, and the controlled nature of studies thus far precludes understanding of whether such benefits can be experienced in everyday life. Nonetheless, the findings indicate an avenue for future research which could make unique contributions to the literature.

## 6. Taste

Taste is a very specific and close-range sense that tells us about what we put in our mouths. As such, it is mostly applicable to food, which is, of course, crucial to our survival. Therefore, taste is a very fundamental sense that has implications for our overt health and ties us inextricably to nature. Since taste is mostly about food, other factors that surround food, such as methods of food production, have important implications for our well-being. Food comes from nature, from local or international farms, and as such, can represent a link to nature even when we do not directly experience it. It is something we cannot avoid, a constant reminder of our natural roots; no matter how much we urbanize, we always have to import food from nature.

### 6.1. Enjoyment of Flavours

One way that the sense of taste can contribute to the benefits we receive from nature is through our enjoyment of the flavours in natural foods. Infants were found to respond positively to sugar solutions and negatively to salty and sour flavours [139,140], showing that an emotional response to flavours is present from birth. Links have been found between our emotions and our neuronal responses to the taste (and smell) of food, and there are regulatory mechanisms that produce feelings of satiety and control our intake of food [141,142]. This is important because it regulates our nutrition intake and is thus an important benefit of the natural property of foods. In fact, “taste is unique among sensory systems in its innate association with mechanisms of reward and aversion” [142]. Specifically, sweet flavours are rewarding, while bitter flavours are aversive [142]. This is important as it tells us what is safe to eat, as many bitter flavours are indicative of the toxic properties which many plants and insects use to deter attack [143,144,145,146,147]. Carnivores are more sensitive to bitter tastes than herbivores and are also less tolerant of the related toxins in plants [148]. Therefore one of the benefits of taste is in telling us what foods are healthy and which are toxic.

A study looking at emotional responses to food found that the most common emotions experienced were satisfaction, enjoyment, and desire, while sadness, anger, and jealousy were experienced the least often [149]. Therefore, taste appears to improve positive affect. It is believed that eating may reduce anxiety and other undesirable emotions [150,151], and this could be a nature benefit of what was once completely natural foods (although now there is “processed” food, most studies use natural foods).

### 6.2. More Natural Food

Organic food has become the “more natural” alternative for consumers. For instance, among the reasons for purchasing organic foods, consumers cited “sensory and emotional appeal” as the second most important one, and indicated that more natural foods are perceived as tasting and smelling better, as well as making the consumer feel better when eaten, invoking feelings of comfort, safety, and tradition [152]. Other studies and a review [153] have also highlighted the importance of better taste in organic food selection [154,155,156,157,158,159,160,161,162]. In a sensory analysis using trained panelists, one study found that organic orange juice, though not organic milk, tastes better than conventional orange juice [163]. One theory suggests that the improved taste could be due to the use of lower yield varieties by organic producers, which tend to taste better [164]. Organic foods are also preferred for a perceived higher nutritional quality and greater health benefits [165,166,167], so contributions to health would be another nature benefit of natural foods. Indeed, a Norwegian study, while correlational, found that those eating a diet high in processed foods had a higher level of anxiety, while those eating a diet with more natural foods had a lower incidence of depression [168]. Among schizophrenics, it was found that fewer males were consuming acceptable levels of fruit and vegetables [169]. A link has been theorized in arctic people between a change in diet from traditional (more natural) foods to a Western diet (more processed foods) and a decline in mental health [170], implying that more natural and traditional foods are better for mental well-being. Natural, unprocessed foods are also better for us physically. “Chronic illnesses and health problems either wholly or partially attributable to [a modern diet] represent by far the most serious threat to public health” [171]. Fruits and vegetables have high nutritional content, and there is a movement in South Korea to maintain aspects of the traditional diet considered to be healthy, such as high vegetable intake [172]. The traditional Okinawan diet is well-recognized for its healthy properties, and is much higher in fruits and vegetables than the modern Western diet [173]. There is concern that our modern diet which depends on agricultural and processed foods is not in line with our biologically adapted diet and that this may be causing many of the new chronic illnesses that are associated with Western civilization [171,174,175,176,177].

### 6.3. Growing Your Own Food

Growing your own food means understanding seasonality and having the experience of gardening, both of which are strong nature experiences. A study by Church et al. [178] showed that those who grow their own food are happier than those who do not, accounting for a large number of possible socio-economic confounding variables, but not demonstrating a causal effect. Food growing has been linked with a variety of benefits, including self-fulfillment, identity affirmation, self-help, and mutual support [179], and growing your own food contributes to food safety and tastier, better quality food [180,181,182]. In fact, wanting better tasting food was the top reason respondents to a study cited for growing their own food [178]. It can also be a very satisfying practice [183], promotes skill development [181,184], and connects one to nature [185]. A final benefit of growing food, and the second most popular reason in the above-mentioned study, was economic savings [178]. Community markets with locally grown food have even been used to promote racial equality, as in the case of “Mo’ Better Foods” in the US [186], indicating that the benefits derived from food and taste can be far reaching, from the individual to the community scale. The expansion of food-growing from a male-dominated practice to a more equal and female-including practice has been studied [187], so it has implications for racial and gender relationships, and the local food movement (growing food locally) has been considered as a social movement [188]. Indeed, food ties us together and its use to bring people together socially is well documented [189,190,191,192,193]. Growing food or otherwise interacting in nature can bring us together socially and provide benefits, such as care farms [194] and nature-assisted therapy programs [195]. All of this suggests that community cohesion is one of the possible benefits of natural food.

While the sensory avenue of taste is the least explored in the literature, there is some indication that growing your own food has beneficial effects, flavours of foods have an effect on emotions, that organic (i.e., more natural foods) are perceived as tasting better and making one feel better, that traditional, more natural diets are better for our physical and mental health, and that growing your own food has positive effects on the individual and on the community.

## 7. Touch

The tactile sense is greatly underappreciated in humans, and its importance is often overlooked. Touch is the first sense to develop in utero [196], and it can affect people’s willingness to comply with requests, create bonds between people and groups, strengthen romantic relationships, and reduce stress as measured by blood pressure and heart rate [197]. Touch is crucial to love and social bonding, and there are many uses of tactile stimulation [198,199]. Contact is very important for humans [200,201,202], and contact comfort is necessary for baby monkeys, without which they become seriously psychologically dysfunctional and deprived [203,204].

In terms of nature experiences, one of the main avenues of experiencing touch is via animal petting. Animals in general have been found to provide us with positive benefits such as reduced blood pressure, self-reported increases in relaxation and comfort, and improved social responses in asocial and autistic individuals [205,206], and contact with animals results in greater benefits than landscape exposure, highlighting the importance of the senses other than vision to nature benefits [207]. Pets increase pain tolerance, decrease loneliness, enhance recovery from stress and frustration, and reduce the need for medical care [208], and while this may be from non-tactile factors such as companionship or increased exercise, animals are known for giving tactile comfort, so this may play a part.

Additionally, there is some literature on the haptic benefits of interacting with nature. This involves taking a “hands on” approach to nature, such as that practiced in forest schools, where children are encouraged to play and learn outdoors; this is believed to be one mechanism through which nature benefits can be delivered [209]. Children at these forest schools are believed to benefit from direct experience with nature in “confidence, social skills, language and communication, motivation and concentration, physical skills, and knowledge and understanding” [210], and motor skills have been found to improve more from play in the forest than in playground areas [211].

### 7.1. Cardiovascular and Mood Effects

Touching animals has been found to have beneficial cardiovascular effects [212,213,214], and it has been found that talking to and petting a dog is less arousing than talking to people [215,216]. Due to the “pet effect” [217], touching dogs can result in lower blood pressure and heart rate than other relaxing activities such as reading [218], and petting a dog lowers stress and decreases salivary and serum cortisol [219]. Arousal may be decreased by touch and this may be because the physical sensation of touch affects the cardiovascular system [217]. According to the “contact comfort hypothesis”, touch is a primary factor in reducing anxiety and sympathetic arousal [217,220]. Interestingly, blood pressure in individuals is affected by petting a dog regardless of their feelings towards animals, so it is not operant conditioning which results in lower pressure, but the act of touching itself [217].

These findings hold true for animals other than dogs as well. Short periods of petting resulted in lower state anxiety scores in stressful situations both with rabbits and with turtles, but petting a soft toy did not show the same results [221]. This was also found to occur independently of attitude towards animals. It appears that touching some living things regardless of attitude towards them results in positive feelings and lower stress, pain, and anxiety [222,223,224].

These benefits also accrue in the long term. Heart disease patients who were pet owners had a higher one-year survival rate [225], and pet owners were found to be healthier than non-pet-owners [226]. Pet owners have lower blood pressure, heart rates [227], and triglyceride levels [228], and petting effects on blood pressure were found to be strongest in individuals with the highest levels of blood pressure [220], so the benefits seem to be greater for those who are most at risk. While these studies of pet-owners are correlational and the effects may be due to factors other than touch (such as increased exercise from dog-walking), they are included here due to the general dearth of studies in the field, and are used to point to useful directions for future studies.

Human-animal interactions activate the oxytocinergic system, resulting in decreased social stress and endocrinological, psychophysiological, and psychosocial effects [229]. Oxytocin is produced by stroking [230,231], and an increase in plasma oxytocin was found in humans after 5–24 min of petting [232,233]. Oxytocin is found to increase social interaction [234], decrease stress [235,236,237], lower pain thresholds, produce anti-inflammatory effects [238,239], lower anxiety [240], and increase the function of the parasympathetic nervous system, resulting in increased digestive function [241,242]. Oxytocin may therefore be an important factor in the nature benefits received from touch.

Animals (for petting and interaction) have been used with psychiatric patients for their beneficial effects and have been shown to decrease fear and anxiety [243]. They are also used in companion animal therapy with outpatient psychiatric children [244]. Children with dog support were found to have lower cortisol levels correlated with their physical contact with the animal [245]. Animal assisted interventions result in lower depression levels [246], and touch was found to decrease cardiovascular activity in hypertensive patients [247]. Although not a treatment, it was found that college students petting live dogs showed an increase in IgA, an indicator of immune system function, indicating that petting animals may also enhance the immune system [248].

### 7.2. Non-Animal Nature Touch

One rather neglected area of research is the health and well-being effects of the non-animal aspects of nature through touch, such as feeling the grass under your feet, the water ripple through your hands, or the wind on your face (which could also be considered thermoception). We could locate no research focused on these topics, suggesting a significant gap in the literature. It would be interesting to look at blood pressure effects, for instance, of lying in the grass, to see if it provides additional benefits to those of just lying down. Part of the pleasure gardeners have in physical contact with the soil may be due to a sensual, touch component.

## 8. Non-Sensory Pathways

There is intriguing emerging evidence of at least three further distinct pathways for benefits of nature experiences that do not fit neatly in one or another of the senses, but that still require contact with nature, and we treat these in the following sections.

### 8.1. Phytoncides

Phytoncides are antimicrobial volatile organic compounds emitted by plants typically for defense against decay or attack by herbivores. Phytoncides permeate the air in natural environments, and are directly ingested by visitors to environments containing plants emitting them. They are not smelled or tasted as such, but simply ingested through inhalation. They are a popular topic of study in Japan, and widely believed to contribute to nature benefits experienced during nature walks known as “shinrin-yoku”, or “forest-bathing” [249].

Two kinds of phytoncides have been found to be antimicrobial on inhalation [250,251,252,253], while three have been found to increase immune system activity in vitro [250]. Overall, phytoncides are believed to decrease stress and increase relaxation, as in rats, they decrease spontaneous activity and reduce cardiovascular response to the stress of restraint [254]. They also prolong sleep, decrease anxiety, and depress the central nervous system in mice [255].

### 8.2. Negative Air Ions

Air ions are positively or negatively charged air particles that form when energy detaches an electron from one gas molecule and attaches it to another [256]. The energy for this ionization comes from radiation, cosmic rays [256], electromagnetic solar waves [257], waterfalls [258] thunder, radiant energy, and UV light [259]. Air ions are particularly abundant in natural places, such as forests and waterfalls, and they have been suggested as one of the potential mechanisms for the physiological and mood benefits of natural places [260]. Built environments tend to be characterized by ion depletion, with the indoors containing as little as 10% of the air ion concentration of the outdoors [261,262,263,264]. With urban populations spending as much as 90% of their time indoors [265], this could be a fundamentally important, yet essentially overlooked pathway for reduced mood and health through reduced contact with nature. Air ions are much more common in outdoor rural air that outdoor urban air, with the former containing in the range of 1200 ions/cm^3^ and the latter about 500 ions/cm^3^ [264]. This may be due to the tendency for small air ions to cluster around pollutants and drop out of the air column [266], and this ability to clean the air is itself a potential benefit of ambient air ions. Vegetation strongly influences the abundance of air ions, with forests having air ion concentrations on average of 1649 ions/cm^3^ in comparison with 494 ions/cm^3^ in open grassy parks as measured in southeast Queensland [267]. Plants directly produce air ions [267,268], and also draw up radon in the groundwater, which is a source of ions [267]. Recent surveys have found that mountains have the highest levels of air ions, while rural and coastal sites have moderate amounts and urban sites have the lowest [269,270].

Air ions have been believed to exert a biological influence since their discovery at the turn of the century [271], and a relationship has been noted between areas of higher air ion concentration (such as mountains and seashores), and areas traditionally prescribed for health treatment [272]. Yet the results of experimentation with air ions have been contradictory, with many experiments being flawed in not assessing the microclimate, improperly measuring ion concentration, and poor preparation of the experimental subject [272], so care should be taken when interpreting the results. There is enough evidence to warrant further exploration, but not enough to draw any firm conclusions.

#### Effects of Negative Air Ions

Air ions kill bacteria, increase plant and insect growth rate, and cause physiological and behavioral changes in people and other animals [273,274,275,276]. About 1/3 of the population is sensitive to air ions, and this portion of the population responds to the change in positive ion concentration that precedes certain warm, dry winds in a number of countries [277] by showing elevated symptoms of depression, lassitude, migraine, nausea, insomnia, and respiratory problems when these winds bring a high concentration of positive ions and a low concentration of negative ions [278,279]. Because negative air ions decrease the concentration of 5-hydroxytryptamine (5-HT) in mice, rabbits and guinea pigs, while positive ions increase it [280,281], and 5-HT in humans has been found to increase with the winds, it is believed that a “serotonin irritation syndrome”, contributes to the symptoms described above [277,282].

Negative air ions, on the other hand, increase thermal comfort and alertness, and decrease stuffiness, nausea, dizziness, and incidence of headaches by 50% in office workers [264]. After exercise, negative air ions decrease serum serotonin [283], and during stress, they decrease immunoreactivity, as well as state-trait anxiety inventory scores and correlate with a slight increase in performance on a word processing task [260]. In addition to a certain proportion of the population being more sensitive to air ions, those under stress or with ailments are also more responsive [284,285], and people with a lower autonomic lability score (more responsive to stress) show a higher response to air ions [286].

High density negative air ions are used as a treatment for seasonal affective disorder [287,288,289], and non-seasonally-depressed individuals showed a 27–55% improvement in depression, mood disturbance, and anger, during the first 30 min of administration of negative air ions in a placebo-controlled study [290]. Negative air ions also increase natural killer cell activity, and have anti-tumor effects [259]. Both positive and negative ions affect alpha brain wave activity, which indicates wakeful relaxation [291,292,293]. Asthma patients showed an 80% improvement using ionized aerosols [294], and ions have been used to improve burn healing [295]. In addition, anxiety was decreased in 80% of patients with anxiety syndrome [296].

In an example from animals, rats show an increase in learning and performance and decrease in fear with negative air ions [297,298,299]. While indoor ion depletion is correlated with depression and somnolence [300] and animals die in air filtered of ions [301,302], increased negative air ions levels stabilize mood, and increase vigor and friendliness [303]. They also increase alertness and performance, decrease tension [304], and increase reaction time, energy, and ease of concentration [305]. In Zhongxiang, a Chinese city known for having long-lived residents, higher levels of air ions were found both indoors and outdoors, a result which, while correlational, is believed to imply that higher levels of air ions contribute to longevity [306]. Negative air ions, therefore, may be an important contributor to the benefits derived from nature and are another possible mechanism through which nature benefits are delivered

### 8.3. Soil and Gut Microbes

In addition to the nutritional value of foods, we ingest a number of microorganisms with our food or directly from the environment, such as from soil. Some of these microorganisms persist as fauna within the gut with a number of beneficial effects. Humans coevolved with microbes for over 500 million years [307,308], and this has led to a symbiotic relationship, wherein bidirectional neuronal, hormonal, and immunological signals are exchanged between the gastrointestinal tract and the brain [309]. Saprophytic (soil) bacteria are commonly found in the gut, and while they cannot replicate there, were present in our ancestors due to exposure through mud and water [310]. Repeated exposure to these organisms was found to lead to a tolerance response to stress [311], and indeed continued exposure to environmental organisms is necessary to maintain the diversity of gut microbiota [312].

The human intestine has 100 trillion bacteria [313], or 10–100 times more bacteria than cells in the human body [313,314,315]. These bacteria come from the soil, water, animal feces, and spores in the air [308,312]. This gut microbiota, consisting of anaerobic bacteria, viruses, protozoa, archae, and fungi [316] is very important to central nervous system function [317,318,319,320,321]. However, the increase in time spent indoors and the sanitization of our living conditions has meant that we are exposed to fewer of these microorganisms than before, and thus reap fewer of the benefits.

#### 8.3.1. Old Friends

It has been suggested that many of the modern world’s chronic diseases are actually inflammatory conditions resulting from the depletion from our environment of the microorganisms with which humans coevolved, the so-called “old friends hypothesis”, also known as the “hygiene hypothesis” [308,322]. These commensal organisms have a role in immunoregulation, and their absence leads to immunological dysregulation, with effects on behavior, emotion, and health [308,311,323]. Commensal microorganisms are also involved in the development of other organ systems, beyond the gut, including bones and the brain [324]. Indeed, microbiota-free mice are found to have altered brain chemistry and stress responses [317,320,325].

#### 8.3.2. Chronic Inflammation and Disease

Urban populations have more mood and anxiety disorders (which are correlated with inflammation) than rural populations [326], and in low income (and therefore more rural) countries, inflammation increases with infection and then decreases afterwards. However, in high income countries, high levels of inflammation are found both with and without infection, such that the level of inflammation never falls [327]. In high income countries, as a result, the rate of chronic inflammatory disorders is high [328], and in both high income and urban areas, chronic inflammation and psychiatric disorders are more common than in low income and rural areas [329]. Overall, then, there is a strong link between gut biodiversity and mental and physical health.

This difference between low income and high income countries and rural and urban residents is exemplified by the difference found in the body microbiota of populations from different countries [312]. A study found that Italians have quite a different gut diversity to that of traditional people from Burkina Faso [330], and the skin microbiota from agricultural residents in Finland was more diverse than that from urban residents [323]. Gut microbe diversity was found to be highest in Amazonian Amerindians, then Malawians, and finally lowest in Americans [331]. People exposed to farm environments while young also have a lower incidence of asthma than the general population [332], and early farm exposure has been considered a protective device since the 19th century [333].

#### 8.3.3. Effects of Microbiota

Commensal organisms produce serotonin, melatonin, gamma-aminobutyric acid, catelcholamines, histamine, and acetylcholine, all neuroactive molecules [308], and so can be expected to affect mood. A soil bacteria, *Mycobacterium vaccae*, has been show to increase emotional affect and cognitive function in cancer patients [334,335,336] by inducing Treg production, which downregulates inflammation [308].

*Mycobacterium vaccae* is an aerobic, temperate bacterium to which we are exposed in water, soil, and vegetation [337,338,339]. As an aerobe, it does not colonize the intestinal tract, but is considered a “transient commensal” [340]. It is believed that *M. vaccae* alters serotonin levels, affecting mood, arousal, and learning [341,342], and in mice, it lowered maze run times, mistakes, and anxiety behaviors [311]. This effect was temporary, only affecting the mice while the bacteria was in their system.

This data on bacterial effects on health indicates that the “old friends hypothesis” could be an avenue through which nature benefits are delivered. It is a rather well studied avenue through which nature benefits are delivered though it has not necessarily been linked with the nature benefits literature. Perhaps some of the benefits derived from living near greenspace [343,344] are due to the spores we ingest and organisms we are exposed to in the air, soil, and water.

## 9. Future Research

Overall, while the visual sense is relatively well understood as a pathway through which the benefits of experiencing nature are delivered, there are substantial deficiencies in our understanding of most of the other sensory and non-sensory pathways. As such, there are many key knowledge gaps and avenues to look into for future research (Table 1). In general, we suggest explicitly looking at senses separately in nature benefits research. This can be done by repeating some experiments but using them on another sense, or on sensory-impaired individuals. Researchers could also build smellscapes and soundscapes to use as model systems, and perhaps couple these with visual virtual reality immersive experiences. One useful way to organize future research in this area is by hypothesizing the pathways leading from nature to the realized benefit and studying key links along this causal pathway [345]. Additionally, more work is needed on the duration of nature benefits, in particular to understand whether short term benefits, which are by far the most commonly studied type of benefit, translate into longer term effects. Other key research agendas include investigating potential synergies of multiple pathways, both sensory and non-sensory, and studying the differences between passive and active sensory avenues.

In the case of hearing, it would be interesting to partition different kinds of natural sounds to determine whether the benefits vary with the acoustic properties of the sounds (e.g., biophysical versus animal sounds or different kinds of bird song). Also, running experiments with visually-impaired individuals, such as exposing them to nature sounds, could provide insight into the nature benefits of sound.

The smell benefits literature could be usefully expanded by focusing on nature smells and the benefits we receive from them, using actual natural products to produce the smells, in the lab and also in situ. Comparisons among negatively and positively-valenced smells to give an idea of smell preference would be interesting, and could start to reveal information about how smells relate to preference and well-being. Finally, self-report studies looking at memories of preferred smells might provide some insight.

Taste remains highly neglected in the context of nature experiences, and some interesting research avenues might include clinical studies on the emotional effects of eating processed versus natural foods, ability to distinguish between processed and natural food, and cognitive effects of diet.

Touch is also rather poorly studied, and many of the references we reviewed were not directly about touch. However, there are intriguing indications that touching animals contributes to health and well-being, and we suggest that this be researched more thoroughly and experimentally where possible. There is also a significant gap in the literature with regards to touching non-animal aspects of nature, such as plants.

Most experiments with phytoncides take place in the lab as this is necessary to isolate the compounds, so an avenue of future research would be to conduct experiments in the field but attempting to isolate phytoncides as the active element. Measurements could also be taken of the distribution of phytoncides in the environment, such as through a park or forest, or in comparison to an urban area. Experiments could also look at how far phytoncides extend from natural areas, and whether they are released in measurable quantities from different kinds of urban greenery.

Because there is so much controversy over whether or not negative air ions actually do have an effect on humans, future research could focus on replicating existing fundamental studies with improved experimental structure and controls. Studies could also look at negative air ion distributions in nature, levels of negative air ions around different kinds of vegetation, and whether negative air ion sensitive individuals receive stronger benefits from experiences of nature.

Microorganisms living in and on people have been heavily studied, but this literature is only just beginning to be linked with the nature benefits literature. The field of microorganisms is quite well studied, but as regards future research, Matthews and Jenks note that, “research that incorporates a behavioral ecological perspective on brain-gut-microbe interactions is necessary” [311], meaning that we need to branch out in the microbiota research into human behavior and human ecology.

## 10. Conclusions

Many benefits that people receive from nature accrue through the five senses as well as at least three non-sensory avenues: sight, sound, smell, taste, touch, phytoncides, negative air ions, and microbes. Most research focuses on the visual nature benefits, and we have briefly reviewed this as well as examining the other pathways through which nature benefits are delivered, concluding that there is a need to broaden work beyond merely the visual sense and to take some experimental studies into the field. The idea that nature provides benefits beyond the visual has been touched on in the literature looking at some mood benefits derived from feeling connected to nature; this suggests that feeling connected to nature is enough to provide some psychological benefits [346,347]. We do not explore this at length here, but this alternate route should not be overlooked. Additionally, there is clear evidence of the benefits of exercising in greenspace or in simulated nature beyond that of exercising in other environments, implying that there may be another pathway involved here, although we do not yet know whether this is through the senses [348,349,350].

We have outlined the evidence that viewing nature both in pictures and through windows can improve health and mood; sounds such as birdsong and nature sounds provide restoration and enhance affect; smells provide numerous physical and psychological benefits; taste affects emotion, and traditional, natural diets have health benefits; petting animals can be very therapeutic; phytoncides can have a positive effect on our immune system; negative air ions affect our physical and mental well-being; and microbiota in the gut and the brain influence each other. We have looked at some sensory and non-sensory avenues, and it is possible that these pathways work in tandem or parallel, either synergistically, additively, or sub-additively.

Some limitations of our review are that it was narrative rather than systematic, and we suggest future studies take a narrower, more systematic approach that could focus on particular health or well-being outcomes, although at this stage it seems the literature would be too sparse for this kind of treatment of most questions. We also used correlational or preference studies in cases where there was little experimental research to show some of the potential sensory pathways for nature benefits, even if they have not been shown unequivocally. Our review focused on the benefits from nature interactions, but future studies could also include risks.

In the hunt for which mechanisms deliver nature benefits, it is easy to overlook the fact that they may, in fact, depend on multiple channels, and over the course of our evolutionary adaptation to natural environments all our senses have presumably become attuned to nature. Our senses often act in tandem, bringing a multitude of benefits at once. Humans are multi-sensory organisms, and we will only build a true picture of the interdependence of our health and well-being on nature once we understand how nature benefits are delivered through the full range of our senses.

## Figures and Tables

**Table 1:** Knowledge gaps.

Pathway	Knowledge Gaps
Sound	Which kinds of nature sounds are important; studies with visually-impaired individuals
Smell	Study of smells emitted directly from plants; in situ studies; how natural smells affect preferences and memory
Taste	Emotional effects of eating natural food; ability to distinguish natural food; cognitive effects of diet
Touch	Non-animal nature touch; effects of petting different kinds of animals; touch-specific studies
Phytoncides	Field studies; documenting fine-scale environmental distribution; how much is released from greenery, variation among plant species
Negative Air Ions	Replicate and improve studies; environmental distribution; release from greenery; correlation between benefits and sensitivity
Microorganisms	Relatively well-researched; connect variation in nature experiences with variation in microbiota

## References

[1] Hartig T., Mitchell R., de Vries S., Frumkin H. (2014). Nature and health. Ann. Rev. Public Health.

[2] Shanahan D.F., Fuller R.A., Bush R., Lin B.B., Gaston K.J. (2015). The health benefits of urban nature: How much do we need?. BioScience.

[3] Burford A. (1969). The Greek Temple Builders at Epidauros.

[4] Thompson C.W. (2011). Linking landscape and health: The recurring theme. Landsc. Urban Plan..

[5] Montford A. (2004). Health, Sickness, and the Friars in the Thirteenth and Fourteenth Centuries.

[6] Olmstead F.L. (1886). Notes on the Plan of Franklin Park and Related Matters.

[7] Stiles A. (2012). Go rest, young man. Monit. Psychol..

[8] Bowler D.E., Buyung-Ali L.M., Knight T.M., Pullin A.S. (2010). A systematic review of evidence for the added benefits to health of exposure to natural environments. BMC Public Health.

[9] Pearson D.G., Craig T. (2014). The great outdoors? Exploring the mental health benefits of natural environments. Front. Psychol..

[10] Gill T. (2014). The benefits of children’s engagement with nature: A systematic literature review. Child. Youth Environ..

[11] Stuster J. (2011). Bold Endeavors: Lessons from Polar and Space Exploration.

[12] Hunter M., Eickhoff S., Pheasant R., Douglas M., Watts G., Farrow T. (2010). The state of tranquility: Subjective perception is shaped by contextual modulation of auditory connectivity. Neuroimage.

[13] Dijk E., Weffers M. Breathe with the ocean: A system for relaxation using combined audio and haptic stimuli. Proceedings of the Special Symposium on Haptic and Audio-Visual Stimuli: Enhancing Experiences and Interaction.

[14] Kjellgren A., Buhrkall H. (2010). A comparison of the restorative effect of a natural environment with that of a simulated natural environment. J. Environ. Psychol..

[15] English Oxford Living Dictionaries. en.oxforddictionaries.com.

[16] Ryff C.D. (1995). Well-being in adult life. Curr. Dir. Psychol. Sci..

[17] Linton M., Dieppe P., Medina-Lara A. (2016). Review of 99 self-report measures for assessing well-being in adults: Exploring dimensions of well-being and developments over time. BMJ Open.

[18] Velarde M., Fry G., Tveit M. (2007). Health effects of viewing landscapes—Landscape types in environmental psychology. Urban For. Urban Green..

[19] Ulrich R. (1979). Visual landscapes and psychological well-being. Landsc. Res..

[20] Moore E. (1981). A prison environment’s effect on health care service demands. J. Environ. Syst..

[21] Ulrich R. (1984). View through a window may influence recovery from surgery. Science.

[22] Laumann K., Garling T., Stormark K. (2001). Rating scale measures of restorative components of environments. J. Environ. Psychol..

[23] Tennessen C., Cimprich B. (1995). Views to nature: Effects on attention. J. Environ. Psychol..

[24] Ulrich R. Health benefits of gardens in hospitals. Proceedings of the Plants for People International Symposium.

[25] Valdez P., Mehrabian A. (1994). Effects of color on emotions. J. Exp. Psychol..

[26] Guilford J., Smith P. (1959). A system of color preferences. Am. J. Psychol..

[27] Jacobs K., Suess J. (1975). Effects of four psychological primary colors on anxiety state. Percept. Motor Skills.

[28] Frank O., Gilovich T. (1988). The dark side of self- and social perception: Black uniforms and aggression in professional sports. J. Personal. Soc. Psychol..

[29] Berman M., Hout M., Kardan O., Hunter M., Yourganov G., Henderson J. (2014). The perception of naturalness correlates with low-level visual features of environmental scenes. PLoS ONE.

[30] Kardan O., Demiralp E., Hout M.C., Hunter M.R., Karimi H., Hanayik T., Yourganov G., Jonides J., Berman M.G. (2015). Is the preference of natural versus man-made scenes driven by bottom-up processing of the visual features of nature?. Front. Psychol..

[31] Aks D., Sprott J. (1996). Quantifying aesthetic preference for chaotic patterns. Empir. Stud. Arts.

[32] Bishop I., Rohrmann B. (2003). Subjective responses to simulated and real environments: A comparison. Landsc. Urban Plan..

[33] Faure P., Hoy R. (2000). The sounds of silence: Cessation of signing and song pausing are ultrasound-induced acoustic startle behaviors in the katydid *Neoconocephalus esige*r (Orthoptera; Tettigoniidae). J. Comp. Physiol. A.

[34] Pijanowski B.C., Farina A., Gage S.H., Dumyahn S.L., Krause B.L. (2011). What is soundscape ecology? An introduction and overview of an emerging new science. Landsc. Ecol..

[35] Kaplan S. (1995). The restorative benefits of nature: Toward an integrative framework. J. Environ. Psychol..

[36] Schafer R. (1994). The Soundscape: Our Sonic Environment and the Tuning of the World.

[37] Torigoe K. Insights taken from three visited soundscapes in Japan. Proceedings of the World Forum for Acoustic Ecology Symposium.

[38] O’Connor P. (2008). The sound of silence: Valuing acoustics in heritage conservation. Geogr. Res..

[39] Halfpenny E. (2010). Pro-environmental behaviors and park visitors: The effect of place attachment. J. Environ. Psychol..

[40] Yang W., Kang J. (2005). Soundscapes and sound preferences in urban squares: A case study in Sheffield. J. Urban Design.

[41] Fisher J.A. (1999). The value of natural sounds. J. Aesthet. Educ..

[42] Carles J.L., Lopez Barrio I., de Lucio J.V. (1999). Sound influence on landscape values. Landsc. Urban Plan..

[43] Zhang M., Kang J. (2007). Towards the evaluation, description, and creation of soundscapes in urban open spaces. Environ. Plan. B Plan. Design.

[44] Irvine K., Devine-Wright P., Payne S.R., Fuller R.A., Painter B., Gaston K.J. (2009). Green space, soundscape and urban sustainability: An interdisciplinary study. Local Environ..

[45] Pilcher E.J., Newman P., Manning R.E. (2009). Understanding and managing experiential aspects of soundscapes at Muir Woods National Monument. Environ. Manag..

[46] Payne S.R. (2013). The production of a perceived restorativeness soundscape scale. Appl. Acoust..

[47] Purcell T., Peron E., Berto R. (2001). Why do preferences differ between scene types?. Environ. Behav..

[48] Van den Berg A., Koole S., van der Wulp N. (2003). Environmental preference and restoration: (How) are they related?. J. Environ. Psychol..

[49] Alvarsson J., Wien S., Nilsson M. (2010). Stress recovery during exposure to nature sound and environmental noise. Int. J. Environ. Res. Public Health.

[50] Bird W. Natural Thinking: Investigating the Links between the Natural Environment, Biodiversity and Mental Health. https://www.rspb.org.uk/Images/naturalthinking_tcm9-161856.pdf.

[51] Diette G., Lechtzin N., Haponik E., Devrotes A., Rubin H. (2003). Distraction therapy with nature sights and sounds reduces pain during flexible bronchoscopy. Chest.

[52] Arai Y.-C., Ushida T., Matsubara T., Shimo K., Ito A., Oshima K. (2008). Intra-operative natural sound decreases salivary amylase activity of patients undergoing inguinal hernia repair under epidural anesthesia. Reg. Anesth. Pain Med..

[53] Ratcliffe E., Gatersleben B., Sowden P.T. (2013). Bird sounds and their contributions to perceived attention restoration and stress recovery. J. Environ. Psychol..

[54] Annerstedt M., Jonsson P., Wallergard M., Johansson G., Karlson B., Grahn P., Hansen A.M., Wahrborg P. (2013). Inducing physiological stress recovery with sounds of nature in a virtual reality forest—Results from a pilot study. Physiol. Behav..

[55] Ogden J.J., Lindburg D.G., Maple T.L. (1993). The effects of ecologically-relevant sounds on zoo visitors. Curator.

[56] Aghaie B., Rejeh N., Heravi-Karimooi M., Ebadi A., Moradin S.T., Vaismoradi M., Jasper M. (2014). Effect of nature-based sound therapy on agitation and anxiety in coronary bypass graft patients during weaning of mechanical ventilation: A randomised clinical trial. Int. J. Nurs. Stud..

[57] Lindquist M., Lange E., Kang J. (2016). From 3D landscape visualization to environmental simulation: The contribution of sound to the perception of virtual environments. Landsc. Urban Plan..

[58] Anderson L., Mulligan B., Goodman L., Regan H. (1983). Effects of sounds on preferences for outdoor settings. Environ. Behav..

[59] Lopez Barrio I., Carles J.L. (1995). Acoustic dimensions of inhabited areas: Quality criteria. Soundscape Newsl..

[60] Rohrmann B., Bishop I. (2002). Subjective responses to computer simulations of urban environments. J. Environ. Psychol..

[61] Mace B.L., Bell P.A., Loomis R.J. (1999). Aesthetic, affective, and cognitive effects of noise on natural landscape assessment. Soc. Nat. Resour..

[62] Mace B.L., Bell P.A., Loomis R.J., Haas G. (2003). Source attribution of helicopter noise in pristine national park landscapes. J. Park Recreat. Adm..

[63] Benfield J., Bell P.A., Troup L., Soderstrom N. (2010). Aesthetic and affective effects of vocal and traffic noise on natural landscape assessment. J. Environ. Psychol..

[64] Kim S.-O., Shelby B. (2011). Effects of soundscapes on perceived crowding and encounter norms. Environ. Manag..

[65] De Coensel B., Vanwetswinkel S., Botteldooren D. (2011). Effects of natural sounds on the perception of road traffic noise. J. Acoust. Soc. Am..

[66] Fisher J.A. (1998). What the hills are alive with: In defense of the sounds of nature. J. Aesthet. Art Crit..

[67] European Information Service (1996). State Aid: Commission Extends Bremer Vulkan Investigation.

[68] European Commission (1996). Commision Green Paper on Future Noise Policy [Com(96)540].

[69] Evans G., Hygge S., Bullinger M. (1995). Chronic noise exposure and psychological stress. Psychol. Sci..

[70] Bronzaft A., Ahern K., McGinn R., O’Connor J., Savino B. (1998). Aircraft noise: A potential hazard. Environ. Behav..

[71] Stansfeld S., Matheson M. (2003). Noise pollution: Non-auditory effect on health. Br. Med. Bull..

[72] Gramann J. (1999). The effect of mechanical noise and natural sound on visitor experiences in units of the National Park Service. Soc. Sci. Res. Rev..

[73] Staples S. (1996). Human response to environmental noise: Psychological research and public policy. Am. Psychol..

[74] Berglund B., Lindvail T., Schwela D., Goh K., World Health Organization (2000). Guidelines for community noise. Guideline Document.

[75] Ohrstrom E. (2004). Longitudinal surveys on effects of changes in road traffic noise—Annoyance, activity disturbances and psycho-social well being. J. Acoust. Soc. Am..

[76] Babisch W., Beule B., Schust M., Kersten N., Ising H. (2005). Traffic noise and risk of myocardial infarction. Epidemiology.

[77] Stansfeld S., Berglund B., Clark C., Lopez Barrio I., Fisher P., Ohrstrom E., Haines M., Head J., Hugge S., van Kamp I. (2005). Aircraft and road traffic noise and children’s cognition and health: A cross-national study. Lancet.

[78] Bluhm G., Berglind N., Nordling E., Rosenlund M. (2007). Road traffic noise and hypertension. Occup. Environ. Med..

[79] Mace B.L., Bell P.A., Loomis R.J. (2004). Visibility and natural quiet in national parks and wilderness areas: Psychological considerations. Environ. Behav..

[80] Gidlof-Gunnarsson A., Ohstrom E. (2007). Noise and well-being in urban residential environments: The potential role of perceived availability to nearby green areas. Landsc. Urban Plan..

[81] (1987). US National Parks. National Parks Overflights Act of 1987. Public Law 100-91 (101 Stat. 674).

[82] (2000). US National Parks. National Parks Air Tour Management Act of 2000. Public Law 106-181, Title VIII (114 Stat. 185).

[83] National Park Service (2006). National Park Service Management Policies.

[84] Bolund P., Hunhammar S. (1999). Ecosystem services in urban areas. Ecol. Econ..

[85] Driver B., Tinsely E., Manfredo M., Brown P., Peterson G. (1991). The paragraphs about leisure and recreation experience preference scales: Results from two inventories designed to assess the breadth of perceived psychological benefits of leisure. Benefits of Leisure Driver.

[86] McDonald C., Baumgartner R., Iachan R. (1995). National Park Service Aircraft Management Studies (USDI Rep. No. 94-2).

[87] Haas G., Wakefield T. (1998). National Parks and the American Public: A National Public Opinion Survey on the National Park System.

[88] Kariel H. (1990). Factors affecting response to noise in outdoor recreational environments. Can. Geogr..

[89] Peretti P.O., Swenson K. (1974). Effects of music on anxiety as determined by physiological skin response. J. Res. Music Educ..

[90] Jordania J. Music and emotions: Humming in human prehistory. In Problems of the Traditional Polyphony Materials of the Fourth International Symposium on Traditional Polyphony.

[91] Spangler H. (1984). Silence as a defense against predatory bats in two species of calling insects. Southwest. Nat..

[92] Dapper A.L., Baugh A.T., Ryan M.J. (2011). The sounds of silence as an alarm cue in tungara frogs, *Physalaemus pustulosus*. BioTropica.

[93] Sherman P. (1977). Nepotism and the evolution of alarm calls. Science.

[94] Seyfarth R., Cheney D., Marier P. (1980). Monkey responses to three different alarm calls: Evidence of predator classification and semantic communication. Science.

[95] Templeton C., Greene E., Davis K. (2005). Allometry of alarm calls: Black capped chickadees encode information about predator size. Science.

[96] Curio E. (1976). The Ethology of Predation.

[97] Glass S.T., Lingg E., Heuberger E. (2014). Do ambient urban odors evoke basic emotions?. Appl. Olfactory Cognit..

[98] Weber S.T., Heuberger E. (2008). The impact of natural odors on affective states in humans. Chem. Sens..

[99] Lorig T.S., Herman K., Schwartz G., Cain W. (1990). EEG activity during administration of low-concentration odors. Bull. Psychon. Soc..

[100] Schloss K.B., Goldberger C.S., Palmer S.E., Levitan C.A. (2015). What’s that smell? An ecological approach to understanding preferences for familiar odors. Perception.

[101] Chen D., Haviland-Jones J. (2000). Human olfactory communication of emotion. Percept. Motor Skills.

[102] Davidson G. (1938). Concerning hallucinations of smell. Psychiatr. Q..

[103] Zald D., Pardo J. (2000). Functional neuroimaging of the olfactory system in humans. Int. J. Psychophysiol..

[104] Kay L.M., Freeman W.J. (1998). Bidirectional processing in the olfactory-limbic axis during olfactory behavior. Behav. Neurosci..

[105] Kohler C., Barrett F., Gur R., Turetsky B., Moberg P. (2007). Association between facial emotion recognition and odor identification in schizophrenia. J. Neuropsychiatry Clin. Neurosci..

[106] Herz R.S., Cupchik G.C. (1995). The emotional distinctiveness of odor-evoked memories. Chem. Sens..

[107] Soudry Y., Lemogne C., Malinvaud D., Consoli S.-M., Bonfils P. (2011). Olfactory system and emotion: Common substrates. Eur. Ann. Ororhinolaryngol. Head Neck Dis..

[108] Horowitz S. (2011). Aromatheraphy: Current and emerging applications. Altern. Complement. Ther..

[109] Price J.L. (1985). Beyond the primary olfactory cortex: Olfactory-related areas in the neocortex, thalamus and hypothalamus. Chem. Sens..

[110] Goel N., Grasso D. (2004). Olfactory discrimination and transient mood change in young men and women: Variation by season, mood state, and time of day. Chronobiol. Int..

[111] Chen D., Haviland-Jones J. (1999). Rapid mood change and human odors. Physiol. Behav..

[112] Lehrner J., Eckersberger C., Walla P., Potsch G., Deecke L. (2000). Ambient odor of orange in a dental office reduces anxiety and improves mood in female patients. Physiol. Behav..

[113] Hermans D., Baeyens F., Eelen P. (1998). Odours as affective-proessing context for word evaluation: A case of cross-modal affective priming. Cognit. Emot..

[114] Millot J.-L., Brand G. (2001). Effects of pleasant and unpleasant ambient odors on human voice pitch. Neurosci. Lett..

[115] Doty R.L. (1986). Odor-guided behavior in mammals. Experientia.

[116] Baron R.A. (1981). Olfaction and human social behavior effects of a pleasant scent on attraction and social perception. Personal. Soc. Psychol. Bull..

[117] Todrank J., Byrnes D., Wrzesniewski A., Rozin P. (1995). Odors can change preferences for people in photographs: A cross-modal evaluative conditioning study with olfactory USs and visual CSs. Learn. Motiv..

[118] Baron R.A. (1997). The sweet smell of... helping: Effects of pleasant ambient fragrance on prosocial behavior in shopping malls. Personal. Soc. Psychol. Bull..

[119] Baron R.A., Thomsley J. (1994). A whiff of reality: Positive affect as a potential mediator of the effects of pleasant fragrances on task performance and helping. Environ. Behav..

[120] Warm J.S., Dember W.N., Parasuraman R. (1991). Effects of olfactory stimulation on performance and stress. J. Soc. Cosmet. Chem..

[121] Smith D.G., Standing L., De Man A. (1992). Verbal memory elicited by ambient odor. Percept. Motor Skills.

[122] Diego M.A., Jones N.A., Field T., Hernandez-Reif M., Schanberg S., Kuhn C., McAdam V., Galamaga R., Galamaga M. (1998). Aromatherapy positively affects mood, EEG patterns of alertness and math computations. Int. J. Neurosci..

[123] Buchbauer G., Jirovetz L., Jager W., Plank C., Dietrich H. (1993). Fragrance compounds and essential oils with sedative effects upon inhalation. J. Pharm. Sci..

[124] Lindsley D., Holmes J. (1984). Basic Human Neuropsychology.

[125] Heuberger E., Hongratanaworakit T., Bohm C., Weber R., Buchbauer G. (2001). Effects of chiral fragrances on human autonomic nervous system parameters and self-evaluation. Chem. Sens..

[126] Haze S., Sakai K., Gozu Y. (2002). Effects of fragrance inhalation on sympathetic activation in normal adults. Jpn. J. Pharmacol..

[127] Kawakami K., Kawamoto M., Nomura M., Otani H., Nabika T., Gonda T. (2004). Effects of phytoncides on blood pressure under restraint stress in SHRSP. Clin. Exp. Pharmacol. Physiol..

[128] Sanderson H., Ruddle J. (1992). Aromatherapy and occupational therapy. Br. J. Occup. Ther..

[129] Valnet R. (1986). The Practice of Aromatherapy.

[130] Conn D., Seiltz D. (2010). Advances in the treatment of psychiatric disorders in long-term care homes. Curr. Opin. Psychiatry.

[131] Hur M., Lee M., Kim C., Ernst E. (2012). Aromatherapy for treatment of hypertension: A systematic review. J. Eval. Clin. Pract..

[132] Cavanagh H., Wilkinson J. (2002). Biological activities of lavendar essential oil. Phytother. Res..

[133] Cooke B., Edzard E. (2000). Aromatherapy—A systematic review. Br. J. Gen.Pract..

[134] Maddocks-Jennings W., Wilkinson J.M. (2004). Aromatherapy practice in nursing—Literature review. J. Adv. Nurs..

[135] Fung J., Tsang H., Chung R. (2012). A systematic review of the use of aromatherapy in treatment of behavioral problems in dementia. Geriatr. Gerontol. Int..

[136] Bakkali F., Averbeck S., Averbeck D., Idaomar M. (2008). Biological effects of essential oils—A review. Food Chem. Toxicol..

[137] Hammer K., Carson C., Riley T. (1999). Antimicrobial activity of essential oils and other plant extracts. J. Appl. Microbiol..

[138] Werker E. (1993). Function of essential oil-secreting glandular hairs in aromatic plants of Lamiacea—A review. Flavour Fragr. J..

[139] Fox N.A., Davidson R.J. (1986). Taste-elicited changes in facial signs of emotion and the asymmetry of brain electrical activity in human newborns. Neuropsychologia.

[140] Crook C. (1978). Taste perception in the newborn infant. Infant Behav. Dev..

[141] Rolls E. (2008). Functions of the orbitofrontal and pregenual cingulate cortex in taste, olfaction, appetite and emotion. Acta Physiol. Hung..

[142] Yamamoto T. (2008). Central mechanisms of taste: Cognition, emotion and taste-elicited behaviors. Jpn. Dent. Sci. Rev..

[143] Wooding S., Gunn H., Ramos P., Thalmann S., Xing C., Meyerhof W. (2010). Genetics and bitter taste responses to goitrin, a plant toxin found in vegetables. Chem. Sens..

[144] Ames B., Profet M., Gold L. (1990). Dietary pesticides (99.99% all natural). Proc. Natl. Acad. Sci. USA.

[145] Bate-Smith E., Harborne J. (1972). Attractants and repellents in higher animals. Phytochemical Ecology: Proceedings of the Phytochemical Society Symposium.

[146] Brieskorn C., Rouseff R. (1990). Physiological and therapeutic aspects of bitter compounds. Bitterness in Foods and Beverages.

[147] Garcia J., Hankins W., Denton D., Coghlan J. (1975). The evolution of bitter and the acquisition of toxiphobia. Olfaction and Taste. V. Proceedings of the 5th International Symposium in Melbourne, Australia.

[148] Glendinning J.I. (1994). Is the bitter rejection response always adaptive?. Physiol. Behav..

[149] Desmet P.M., Schifferstein H.N. (2008). Sources of positive and negative emotions in food experience. Appetite.

[150] Lyman B. (1982). A Psychology of Food, More Than a Matter of Taste.

[151] Canetti L., Bachar E., Berry E.M. (2002). Food and emotion. Behav. Process..

[152] Lockie S., Lyons K., Lawrence G., Grice J. (2004). Choosing organics: A path analysis of factors underlying the selection of organic food among Australian consumers. Appetite.

[153] Hughner R.S., McDonagh P., Prothero A., Schultz C.J., Stanton J. (2007). Who are organic food consumers? A compilation and review of why people purchase organic food. J. Consum. Behav..

[154] Roddy G., Cowan C., Hutchinson G. (1996). Irish market. Br. Food J..

[155] Schifferstein H., Oude Ophuis P. (1998). Health-related determinants of organic food consumption in The Netherlands. Food Q. Preference.

[156] Magnusson M., Arvola A., Hursti U., Aberg L., Sjoden P. (2001). Attitudes towards organic foods among Swedish consumers. Br. Food J..

[157] Lea E., Worsley T. (2005). Australians’ organic food beliefs, demographics and values. Br. Food J..

[158] Institute of Food Science and Technology Organic Food. http://www.ifst.org/.

[159] Roddy G., Cowan C., Hutchinson G. (1996). Consumer attitudes and behaviour to organic foods in Ireland. J. Int. Consum. Mark..

[160] Farah A.S., Rennie D. (2012). Consumer perceptions towards organic food. Procedia Soc. Behav. Sci..

[161] McEachern M.G., McClean P. (2002). Organic purchasing motivations and attitudes: Are they ethical?. Int. J. Consum. Stud..

[162] Hjelmar U. (2011). Consumers’ purchase of organic food products. A matter of convenience and reflexive practices. Appetite.

[163] Fillion L., Arazi S. (2002). Does organic food taste better? A claim substantiation approach. Nutr. Food Sci..

[164] Davies A., Titterington A.J., Cochrane C. (1995). Who buys organic food? A profile of the purchasers of organic food in Northern Ireland. Br. Food J..

[165] Baker S., Thompson K.E., Engelken J., Huntley K. (2004). Mapping the values driving organic food choice: Germany vs. the UK. Eur. J. Mark..

[166] Zanoli R., Naspetti S. (2002). Consumer motivations in the purchase of organic food: A means-end approach. Br. Food J..

[167] Yiridoe E.K., Bonti-Ankomah S., Martin R.C. (2005). Comparison of consumer perceptions and preference toward organic versus conventionally produced foods: A review and update of the literature. Renew. Agric. Food Syst..

[168] Jacka F.N., Mykletun A., Berk M., Bjelland I., Tell G.S. (2011). The association between habitual diet quality and the common mental disorders in community-dwelling adults: The Hordaland health study. Psychosom. Med..

[169] McCreadie R.G. (2003). Diet, smoking and cardiovascular risk in people with schizophrenia: Descriptive study. Br. J. Psychiatry.

[170] McGrath-Hanna N.K., Greene D.M., Tavernier R.J., Bult-Ito A. (2003). Diet and mental health in the Arctic: Is diet and important risk factor for mental health in circumpolar peoples? A review. Int. J. Circumpolar Health.

[171] Cordain L., Eaton S.B., Sebastian A., Mann N., Lindeberg S., Watkins B.A., O’Keefe J.H., Brand-Miller J. (2005). Origins and evolution of the Western diet: Health implications for the 21st century. Am. J. Clin. Nutr..

[172] Lee M.-J., Popkin B.M., Kim S. (2002). The unique aspects of the nutrition transition in South Korea: The retention of healthful elements in their traditional diet. Public Health Nutr..

[173] Willcox D.C., Willcox B.J., Todoriki H., Suzuki M. (2009). The Okinawan diet: Health implications of a low-calorie, nutrient-dense, antioxidant-rich dietary pattern low in glycemic load. J. Am. Coll. Nutr..

[174] Boaz N. (2002). Evolving Health: The Origins of Illness and How the Modern World Is Making Us Sick.

[175] Nesse R., Williams G. (1994). Why We Get Sick. The New Science of Darwinian Medicine.

[176] Eaton S.B., Konnor M. (1985). Paleolithic nutrition. A consideration of its nature and current implications. N. Engl. J. Med..

[177] Eaton S.B., Konner M., Shostak M. (1988). Stone agers in the fast lane: Chronic degenerative diseases in evolutionary perspective. Am. J. Med..

[178] Church A., Mitchell R., Ravenscroft N., Stapleton L. (2015). ‘Growing your own’: A multi-level modelling approach to understanding personal food growing trends and motivations in Europe. Ecol. Econ..

[179] Crouch D., Ward C. (1999). The Allotment: Its Landscape and Culture.

[180] National Gardening Association The Impact of Home and Community Gardening in America. http://www.gardenresearch.com/files/2009-Impact-of-Gardening-in-America-White-Paper.pdf.

[181] Kortright R., Wakefield S. (2011). Edible backyards: A qualitative study of household food growing and its contributions to food security. Agric. Hum. Values.

[182] Wakefield S., Yeudall F., Taron C., Reynolds J., Skinner A. (2007). Growing urban health: Community gardening in South East Toronto. Health Promot. Int..

[183] Tomkins M. (2014). Making Space for Food: Everyday Community Food Gardening and Its Contribution to Urban Agriculture.

[184] Clavin A. (2011). Realising ecological sustainability in community gardens: A capability approach. Local Environ..

[185] Fuller R.A., Irvine K.N. (2010). Interactions between people and nature in urban environments. Urban Ecology.

[186] Alkon A.H. (2007). Growing resistance: Food, culture and the mo’ better foods farmers’ market. Gastronomica.

[187] Buckingham S. (2005). Women (re)construct the plot: The regen(d)eration of urban food growing. Area.

[188] Starr A. (2010). Local food: A social movement?. Cult. Stud. Crit. Methodol..

[189] Johnson M. (1986). Food and Culture Among Bolivia Aymara. Symbolic Expressions of Social Relations.

[190] Rogers G.A. (1975). Kai and Kava in Niutoputapu: Social Relations, Ideologies and Contexts in a Rural Tongan Community.

[191] Testart A., Forbis R.G., Hayden B., Ingold T., Perlman S.M., Pokotylo D.L., Rowley-Conway P., Stuart D.E. (1982). The significance of food storage among hunter-gatherers: Residence patterns, population densities, and social inequalities. Curr. Anthropol..

[192] Counihan C.M. (1984). Bread as world: Food habits and social relations in modernizing Sardinia. Anthropol. Q..

[193] Connor M., Armitage C.J., Conner M. (2002). The Social Psychology of Food.

[194] Hine R., Peacock J., Pretty J. (2008). Care farming in the UK: Contexts, benefits, and links with therapeutic communities. Ther. Commun..

[195] Annerstedt M., Wahrborg P. (2011). Nature-assisted therapy: Systematic review of controlled and observational studies. Scand. J. Soc. Med..

[196] Dijk E., Nijholt A., van Erp J.B., Kuyper E., van Wolferen G. Audio-tactile stimuli to improve health and well-being: A preliminary position paper. Proceedings of the Symposium on Haptic and Audio-Visual Stimuli: Enhancing Experiences and Interaction.

[197] Gallace A., Spence C. (2010). The science of interpersonal touch: An overview. Neurosci. Biobehav. Rev..

[198] Essick G.K., McGlone F., Dancer C., Fabricant D., Ragin Y., Phillips N., Jones T., Guest S. (2010). Quantitative assessment of pleasant touch. Neurosci. Biobehav. Rev..

[199] Dunbar R.I. (2010). The social role of touch in humans and primates: Behavioural function and neurobiological mechanisms. Neurosci. Biobehav. Rev..

[200] Moss H., Spencer T., Kass N. (1970). Early environmental effects: Mother-child relations. Perspectives in Child Psychology.

[201] Passman R. (1977). Providing attachment objects to facilitate learning and reduce distress: Effects of mothers and security blankets. Dev. Psychol..

[202] Schaffer H., Emerson P. (1964). The development of social attachment in infancy. Monogr. Soc. Res. Child Dev..

[203] Harlow H. (1962). The heterosexual affectional system in monkeys. Am. Psychol..

[204] Harlow H., Zimmerman R. (1959). Affectional responses in the infant monkey. Science.

[205] Katcher A., Wilkins G., Kellert S., Wilson E. (1993). Dialogue with animals: Its nature and culture. The Biophilia Hypothesis.

[206] Shepard P. (1996). The Others: How Animals Made Us Human.

[207] Kahn P.H.J. (1997). Developmental psychology and the biophilia hypothesis: Children’s affiliation with nature. Dev. Rev..

[208] Kellert S., Wilson E. (1993). The Biophilia Hypothesis.

[209] O’Brien L., Burls A., Brensten P., Hilmo I., Holter K., Haberling D., Pirnat J., Sarv M., Vilbaste K., McLoughlin J. (2011). Outdoor education, life long learning and skills development in woodlands and green spaces: The potential links to health and well-being. Forests, Trees and Human Health.

[210] O’Brien L., Murray R. (2007). Forest school and its impact on young children: Case studies in Britain. Urban For. Urban Green..

[211] Fjortoft I. (2001). The natural environment as a playground for children: The impact of outdoor play activities in pre-primary school children. Early Child. Educ. J..

[212] Friedmann E., Katcher A., Thomas S., Lynch J., Messent P. (1983). Social interaction and blood pressure influence of animal companions. J. Nerv. Ment. Dis..

[213] Grossberg J., Alf E. (1985). Interaction with pet dogs: Effects on human cardiovascular response. J. Delta Soc..

[214] Jenkins J. (1986). Physiological effects of petting a companion animal. Psychol. Rep..

[215] Friedmann E., Katcher A., Meislich D., Goodman M. (1979). Physiological response of people to petting their pets. Am. Zool..

[216] Katcher A., Fogle B. (1981). Interactions between people and their pets: Form and function. Interrelations between People and Pets.

[217] Vormbrock J.K., Grossberg J.M. (1988). Cardiovascular effects of human-pet dog interactions. J. Behav. Med..

[218] Wilson C. (1987). Physiological responses of college students to a pet. J. Nerv. Ment. Dis..

[219] Barker S., Knisley J., McCain N., Best A. (2005). Measuring stress and immune responses in health care professionals following interaction with a therapy dog: A pilot study. Psychol. Rep..

[220] Friedmann E., Thomas S. (1985). Health benefits of pets for families. Marriage Fam. Rev..

[221] Shiloh S., Sorek G., Terkel J. (2003). Reduction of state-anxiety by petting animals in a controlled laboratory experiment. Anxiety Stress Coping Int. J..

[222] Lafreniere K., Mutus B., Cameron S., Tannous M., Gianotti M., Abu-Zahra H., Laukkanen E. (1999). Effects of therapeutic touch on biochemical and mood indicators in women. J. Altern. Complement. Med..

[223] Montagu A. (1978). Touching.

[224] Spence J., Olson M. (1997). Quantitative research on therapeutic touch—An integrative review of the literature 1985–1995. Scand. J.Caring Sci..

[225] Friedmann E., Katcher A., Lynch J., Thomas S. (1980). Animal companions and one year survival after discharge from a coronary care unit. Public Health Rep..

[226] Headey B., Grabka M. (2007). Pets and human health in Germany and Australia: National longitudinal results. Soc. Indic. Res..

[227] Allen K., Blascovich J., Mendes W. (2002). Cardiovascular reactivity and the presence of pets, friends, and spouses: The truth about cats and dogs. Psychosom. Med..

[228] Anderson W.P., Reid C.M., Jennings G.L. (1992). Pet ownership and risk factors for cardiovascular disease. Med. J. Aust..

[229] Beetz A., Uvnas-Moberg K., Julius H., Kotrschal K. (2012). Psychosocial and psychophysiological effects of human-animal interactions: The possible role of oxytocin. Front. Psychol..

[230] Uvnas-Moberg K. (2003). The Oxytocin Factor. Tapping the Hormone of Calm, Love, and Healing.

[231] Insel T. (2010). The challenge of translation in social neuroscience: A review of oxytocin, vasopressin, and affiliative behavior. Neuron.

[232] Odendaal J. (2000). Animal-assisted therapy—Magic or medicine?. J. Psychosom. Res..

[233] Odendaal J., Meintjes R. (2003). Neurophysiological correlates of affiliative behavior between humans and dogs. Vet. J..

[234] Ditzen B., Schaer M., Gabriel B., Bodenmann G., Ehlert U., Heinrichs M. (2009). Intranasal oxytocin increases positive communication and reduces cortisol levels during couple conflict. Biol. Psychiatry.

[235] Legros J., Chiodera P., Geenen V. (1988). Inhibitory action of exogenous oxytocin on plasma cortisol in normal human subejcts: Evidence of action at the adrenal gland. Neuroendocrinology.

[236] Petersson J., Lundeberg T., Uvnas-Moberg K. (1999). Short-term increase and long-term decrease of blood pressure in response to oxytocin-potentiating effect of female steroid hormones. J. Cardiovasc. Pharmacol..

[237] Neumann I., Wigger A., Torner L., Holsboer F., Landgraf R. (2000). Brain oxytocin inhibits basal and stress-induced activity of the hypothalamo-pituitary-adrenal axis in male and female rats: Partial action within the paraventricular nucleus. J. Neuroendocrinol..

[238] Petersson M., Alster P., Lundeberg T., Uvnas-Moberg K. (1996). Oxytocin increases nociceptive thresholds in a long-term perspective in female and male rats. Neurosci. Lett..

[239] Petersson M., Eklund M., Uvnas-Moberg K. (2005). Oxytocin decreases corticosterone and nociception and increases motor activity in OVX rats. Maturitas.

[240] Neumann I., Kromer S., Toschi N., Ebner K. (2000). Brain oxytocin inhibits the (re)activity of the hypothalamo-pituitary-adrenal axis in male rats: Involvement of hypothalamic and limbic brain regions. Regul. Pept..

[241] Widstrom A., Winberg J., Werner S., Svensson K., Poloncec B., Uvnas-Moberg K. (1988). Breast feeding-induced effects on plasma gastrin and somatostatin levels and their correlation with mild yield in lactating females. Early Hum. Dev..

[242] Uvnas-Moberg K. (1989). The gastrointestinal tract in growth and reproduction. Sci. Am..

[243] Barker S., Pandurangi A., Best A. (2003). Effects of animal-assisted therapy on patients’ anxiety, fear, and depression before ECT. J. ECT.

[244] Levinson B. (1969). Pet Oriented Child Psychotherapy.

[245] Beetz A., Kotrschal K., Hediger K., Turner D., Uvnas-Moberg K. (2011). The effect of a real dog, toy dog and friendly person on insecurely attached children during a stressful task: An exploratory study. Anthrozoos.

[246] Souter M., Miller M. (2007). Do animal-assisted activities effectively treat depression? A meta-analysis. Anthrozoos.

[247] Allen K., Shykoff B., Izzo J. (2001). Pet ownership, but not ACE inhibitor therapy, blunts blood pressure responses to mental stress. Hypertension.

[248] Charnetski C., Riggers S., Brennan F. (2004). Effect of petting a dog on immune system function. Psychol. Rep..

[249] Li Q. (2010). Effect of forest bathing trips on human immune function. Environ. Health Prev. Med..

[250] Li Q., Nakadai A., Matshushima H., Miyazaki Y., Krensky A., Kawada T. (2006). Phytoncides (wood essential oils) induce human natural killer cell activity. Immunopharmacol. Immunotoxicol..

[251] Li Q., Morimoto K., Nakadai A., Inagaki H., Katsumata M., Shimizu T. (2007). Forest bathing enhances human natural killer activity and expression of anti-cancer proteins. Int. J. Immunopathol. Pharmacol..

[252] Li Q., Morimoto K., Kobayashi M., Inagaki H., Katsumata M., Hirata Y. (2008). Visiting a forest, but not a city, increases human natural killer activity and expression of anti-cancer proteins. Int. J. Immunopathol. Pharmacol..

[253] Li Q., Morimoto K., Kobayashi M., Inagaki H., Katsumata M., Hirata Y. (2008). A forest bathing trip increases human natural killer activity and expression of anti-cancer proteins in female subjects. J. Biol. Regul. Homeost. Agents.

[254] Kawamoto M., Kawakami K., Otani H. (2008). Effects of phytoncides on spontaneous activities and sympathetic stress responses in Wistar Kyoto and stroke-prone spontaneously hypertensive rats. Shimane J. Med. Sci..

[255] Cheng W.-W., Lin C.-T., Chu F.-H., Chang S.-T., Wang S.-Y. (2009). Neuropharmacological activities of phytoncide released from *Cryptomeria japonica*. J. Wood Sci..

[256] Hawkins L., Barker T. (1978). Air ions and human performance. Ergonomics.

[257] Hansell C. An attempt to define ionisation of the air. Proceedings of the International Conference on Ionisation of the Air.

[258] Chalmers J. (1957). Atmospheric Electricity.

[259] Yamada R., Yanoma S., Akaike M., Tsuburaya A., Sugimasa Y., Takemiya S., Imada T. (2006). Water-generated negative air ions activate NK cell and inhibit carcinogenesis in mice. Cancer Lett..

[260] Nakane H., Asami O., Yamada Y., Ohira H. (2002). Effect of negative air ions on computer operation, anxiety and salivary chromogranin A-like immunoreactivity. Int. J. Psychophysiol..

[261] Anderson I. (1965). The influence of electrical fields on uptake of light gases in a model of man. Int. J. Biometeorol..

[262] Maczynski B., Tyczka S., Mearecki B., Gora T. (1971). Effect of the presence of man on the air ion density in an office room. Int. J. Biometeorol..

[263] Kornblueh I., Swope S., Davis F. Natural ion levels in enclosed spaces. In Proceeding of the Congress on Lacustrine Climatology.

[264] Hawkins L. (1981). The influence of air ions, temperature and humidity on subjective wellbeing and comfort. J. Environ. Psychol..

[265] Leech J., Burnett R., Nelson W., Aaron S., Raizenne M. (2000). Outdoor air pollution epidemiologic studies. Am. J. Respir. Crit. Care.

[266] Ling X., Jayaratne R., Morawska L. (2010). Air ion concentration in various urban outdoor environments. Atmos. Environ..

[267] Jayaratne R., Ling X., Morawska L. (2011). Role of vegetation in enhancing radon concentration and ion production in the atmosphere. Environ. Sci. Technol..

[268] Nemeryuk G. (1970). Salt migration into atmosphere during transpiration. Sov. Plant Physiol..

[269] Pawar S.D., Meena G., Jadhav D. (2012). Air ion variation at poultry-farm, coastal, mountain, rural and urban sites in India. Aerosol Air Q. Res..

[270] Mandija F., Bushati J. Overview of measurements of air ion concentration under specific situations. Proceedings of the ARSA-Advanced Research in Scientific Areas.

[271] Davis J. (1963). Review of scientific information on the effects of ionized air on human beings and animals. Aerosp. Med..

[272] Yates A., Gray F., Misiaszek J., Wolman W. (1986). Air ions: Past problems and future directions. Environ. Int..

[273] Krueger A., Andriese P., Kotaka S. (1968). Small air ions: Their effect on blood levels of serotonin in terms of modern physical theory. Int. J. Biometeorol..

[274] Krueger A. (1982). Air ions as biological agents—Fact or fancy, part I and II. Immol. Allergy Pract..

[275] Kellogg E.I. (1984). Air ions: Their possible biological significance and effects. Electromagn. Biol. Med..

[276] Fornof K., Gilbert G. (1988). Stress and physiological, behavioral and performance patterns of children under varied air ion levels. Int. J. Biometeorol..

[277] Sulman F., Danon A., Pfeifer Y., Tal E., Weller C. (1970). Urinalysis of patients suffering from climatic heat stress (Sharav). Int. J. Biometeorol..

[278] Sulman F. (1971). Meteorological front movements and weather sensitivity. Arztliche Prax..

[279] Krueger A. (1973). Are negative ions good for you?. New Sci..

[280] Krueger A., Smith R. (1960). The biological mechanisms of air ion action II, negative ion effects on the concentration and metabolism of 5-hydroxytryptamine in the mammalian respiratory tract. J. Gen. Physiol..

[281] Krueger A. (1975). Are Air Ions Biologically Active? In Conference on Electrostatics.

[282] Sulman F., Pfeifer Y., Superstine E. (1973). Adrenal medullary exhaustion from tropical winds and its management. Isr. J. Med. Sci..

[283] Ryushi T., Kita I., Sakurai T., Yasumatsu M., Isokawa M., Aihara Y., Hama K. (1998). The effect of exposure to negative air ions on the recovery of physiological responses after moderate endurance exercise. Int. J. Biometeorol..

[284] Sulman F. (1974). Meteorological front movements and human weather sensitivity. Karger Gaz..

[285] Frey A., Granda R. Human reactions to air ions: Experimental controls. Proceedings of the International Conference of Ionization of Air.

[286] Charry J.M., Hawkinshire F.B.V. (1981). Effects of atmospheric electricity on some substrates of disordered social behavior. J. Personal. Soc. Psychol..

[287] Terman M., Terman J. (1995). Treatment of seasonal affective disorder with a high-output negative ionizer. J. Altern. Comlement. Med..

[288] Terman M., Terman J., Ross D. (1998). A controlled trial of timed bright light and negative air ionization for treatment of winter depression. Arch. Gen. Psychiatry.

[289] Goel N., Terman M., Terman J., Macchi M., Stewart J. (2005). Controlled trial of bright light and negative air ions for chronic depression. Psychol. Med..

[290] Goel N., Etwaroo G.R. (2006). Bright light, negative air ions and auditory stimuli produce rapid mood changes in a students population: A placebo-controlled study. Psychol. Med..

[291] Assael M., Pfeifer Y., Sulman F. (1974). Influence of artificial air ionization on the human electroencephalogram. Int. J. Biometeorol..

[292] Silverman D., Kornblueh I. (1957). Effect of artificial ionization of the air on electroencephalogram. Am. J. Phys. Med..

[293] Sulman F.G., Levy D., Lunkan L., Pfeifer Y., Tal E. (1978). Absence of harmful effects of protracted negative air ionization. Int. J. Biometeorol..

[294] Wehner A. (1962). Special review—Electro-aerosol therapy. Am. J. Phys. Med..

[295] Kornblueh I., Gualtierotti R., Kornblueh I., Sirtori E. (1968). Aeroionotherapy of burns. Bioclimatology, Biometeorology and Aeroionotherapy.

[296] Ucha Udabe R., Kertesc R., Franceschetti L., Gualtierotti R., Kornblueh I., Sirtori E. (1968). Use of negative ions in illnesses of the nervous system. Bioclimatology, Biometeorology and Aeroionotherapy.

[297] Frey A. (1967). Modification of the conditioned emotional response by treatment with small negative air ions. J. Comp. Physiol. Psychol..

[298] Nazzaro J., Jackson D., Perkins L. (1967). Effects of ionized air on stress behavior. Med. Res. Eng..

[299] Olivereau J., Lambert J. (1981). Effects of ions on some aspects of learning and memory of rats and mice. Int. J. Biometeorol..

[300] Kimura S., Ashiba M., Matsushima J. (1939). Influences of the air lacking light ions and the effect of its artificial ionization upon human beings in occupied rooms. Jpn. J. Med. Sci. Biol..

[301] Chizhevsky A. (1995). On the Shore of the Universe. Unexplainable Phenomenon.

[302] Goldstein N., Archavskaya T. (1997). Is atmospheric superoxide vitally necessary? Accelerated death of animals in a quasi-neutral electric atmosphere. Z. Naturforsch.

[303] Sigel S. (1979). Bio-Psychological Influences of Air Ions in Men: Effects on 5-Hydroxytryptoamine (5HT) and Mood.

[304] Soyka F., Edwards A. (1976). The Ion Effect.

[305] Tom G., Poole M., Galla J., Berrier J. (1981). The influence of negative air ions on human performance and mood. Hum. Factors.

[306] Lv J., Wang W., Krafft T., Li Y., Zhang F., Yuan F. (2010). Effects of several environmental factors on longevity and health of the human population of Zhongxiang, Hubei, China. Biol. Trace Elem. Res..

[307] Strachan D. (1989). Hay fever, hygeine, and household size. Br. Med. J..

[308] Rook G., Raison C.L., Lowry C.A. (2012). Can we vaccinate against depression?. Drug Discov. Today.

[309] Grenham S., Clarke G., Cryan J., Dinan T. (2011). Brain-gut-microbe communication in health and disease. Front. Physiol..

[310] Rook G., Rook G. (2010). The changing microbial environment, Darwinian medicine and the hygeine hypothesis. The Hygeine Hypothesis and Darwinian Medicine.

[311] Matthews D.M., Jenks S.M. (2013). Ingestion of *Myobacterium vaccae* decreases anxiety-related behavior and improves learning in mice. Behav. Process..

[312] Rook G., Raison C., Lowry C. (2014). Microbial ‘old friends’, immunoregulation and socioeconomic status. Clin. Exp. Immunol..

[313] Gil S.R., Pop M., DeBoy R.T., Eckburg P.B., Turnbaugh P.J., Samuel B.S., Gordon J.I., Relman D.A., Fraser-Liggett C.M., Nelson K.E. (2006). Metagenomic analysis of the human distal gut microbiome. Science.

[314] Luckey T. (1972). Introduction to intestinal microecology. Am. J. Clin. Nutr..

[315] Qin J., Li R., Raes J., Arumugam M., Burgdorf K., Manichanh C. (2010). A human gut microbial gene catalogue established by metagenomic sequencing. Nature.

[316] Xu J., Mahowald M., Ley R., Lozupone C., Hamady M., Martens E. (2007). Evolution of symbiotic bacteria in the distal human intestine. PLoS Biol..

[317] Heijtz R., Wang S., Anuar F., Qian Y., Bjorkholm B., Samuelsson A., Hibberd M., Frossberg H., Pettersson S. (2011). Normal gut microbiota modulates brain development and behavior. Proc. Natl. Acad. Sci. USA.

[318] Neufeld K., Kang N., Bienenstock J., Foster J. (2011). Effects of intestinal microbiota on anxiety-like behavior. Commun. Integr. Biol..

[319] Neufeld K., Kang N., Bienenstock J., Foster J. (2011). Reduced anxiety-like behavior and central neurochemical change in germ-free mice. Neurogastroenterol. Motil..

[320] Sudo N., Chida Y., Aiba Y., Sonoda J., Oyama N., Yu X., Kubo C., Koga Y. (2004). Postnatal microbial colonization programs the hypothalamic-pituitary-adrenal system for stress response in mice. J. Physiol..

[321] Clarke G., Grenham S., Scully P., Fitzgerald P., Moloney R., Shanahan F., Dinan T., Cryan J.F. (2012). The microbiome-gut-brain axis during early life regulates the hippocampal serotonergic system in a sex-dependent manner. Mol. Psychiatry.

[322] Rook G., Brunet L. (2002). Give us this day our daily germs. Biologist.

[323] Hanski I., von Hertzen L., Fyhrquist N., Koskinen K., Torppa K., Laatikainen T., Karisola P., Auyinen P., Paulin L., Makela M.J. (2012). Environmental biodiversity, human microbiota, and allergy are interrelated. Proc. Natl. Acad. Sci. USA.

[324] McFall-Ngai M., Hadfield M., Bosch T. (2013). Animals in a bacterial world, a new imperative for the life sciences. Proc. Natl. Acad. Sci. USA.

[325] Bailey M., Dowd S., Galley J., Hufnagle A., Allen R., Lyte M. (2011). Exposure to a social stressor alters the structure of the intestinal microbiota: Implications for stressor-induced immunomodulation. Brain Behav. Immun..

[326] Peen J., Schoevers R., Beekman A., Dekker J. (2010). The current status of urban-rural differences in psychiatric disorders. Acta Psychiatr. Scand..

[327] McDade T.W., Tallman P.S., Madimenos F.C., Liebert M.A., Cepon T.J., Sugiyama L.S., Snodgrass J.J. (2012). Analysis of variability of high sensitivity C-reactive protein in lowland Ecuador reveals no evidence of chronic low-grade inflammation. Am. J. Hum. Biol..

[328] Bach J. (2002). The effect of infections on susceptibility to autoimmune and allergic diseases. N. Engl. J. Med..

[329] Rook G., Lowry C., Raison C.L. (2013). Microbial old friends, immunoregulation and stress resilience. Evol. Med. Public Health.

[330] De Filippo C., Cavalieri D., Di Paola M., Ramazzotti M., Poullet J.B., Massart S., Collini S., Pieraccini G., Lionetti P. (2010). Impact of diet in shaping gut microbiota revealed by a comparative study in children from Europe and Rural Africa. Proc. Natl. Acad. Sci. USA.

[331] Yatsunenko T., Rey F.E., Manary M.J., Trehan I., Dominguez-Bello M.G., Contreras M., Margris M., Hidalgo G., Baldassano R.N., Anokhin A.P. (2012). Human gut microbiome viewed across age and geography. Nature.

[332] Ege M.J., Mayer M., Normand A.-C., Genuneit J., Crookson W.O., Braun-Fahrlander C., Heederik D., Piarroux R., von Mutius E. (2011). Exposure to environmental microorganisms and childhood asthma. N. Engl. J. Med..

[333] Blackley C. (1873). Experimental Researches on the Causes and Nature of Catarrhus Aestivus (Hay-Fever and Hay-Asthma).

[334] Dalbeth N., Yeoman S., Dockerty J., Highton J., Robinson E., Tan P., Herman D., McQueen F. (2004). A randomised placebo controlled trial of delipidated, deglycolipidated *Mycobacterium vaccae* as immunotherapy for psoriatic arthritis. Ann. Rheum. Dis..

[335] O’Brien M., Anderson H., Kaukel E., O’Byrne K., Pawlicki M., Von Pawel J., Reck M. (2004). SRL172 (killed *Mycobacterium vaccae*) in addition to standard chemotherapy improves quality of life without affecting survival, in patients with advanced non-small-cell lung cancer: Phase III results. Ann. Oncol..

[336] O’Brien M., Saini A., Smith I., Webb A., Gregory K., Mendes R., Ryan C., Priest K., Bromelow K.V., Palmer R.D. (2000). A randomized phase II study of SRL 172 (*Mycobacterium vaccae*) combined with chemotherapy in patients with advanced inoperable non-small-cell lung cancer and mesothelioma. Br. J. Cancer.

[337] Sneath P., Mair N., Sharpe M., Holt J. (1986). Bergey’s Manual of Systematic Bacteriology.

[338] Gomez A., Mve-Obiang A., Vray B., Rudnicka W., Shamputa I., Portaels F., Meyers W., Fonteyne P.-A., Realini L. (2001). Detection of phospholipase in nontuberculous mycobacteria and its possible role in hemolytic activity. J. Clin. Microbiol..

[339] Kazda J., Pavlik I., Falkinham J., Hruska K. (2010). The Ecology of Mycobacteria: Impact on Animals and Human’s Health.

[340] Rook G., Brunet L. (2005). Microbes, immunoregulation and the gut. Gut.

[341] Leussis M., Bolivar V. (2006). Habituation in rodents: A review of behavior neurobiology and genetics. Neurosci. Biobehav. Rev..

[342] Cools R., Roberts A., Robbins T. (2007). Serotonergic regulation of emotion and behavioural control responses. Trends Cognit. Sci..

[343] Maas J., Verheij R., Groenewegen P., de Vries S., Spreeuwenberg P. (2006). Green space, urbanity, and health: How strong is the relation?. J. Epidemiol. Community Health.

[344] Mitchell R., Popham F. (2008). Effect of exposure to natural environment on health inequalities: An observational population study. Lancet.

[345] Shanahan D.F., Lin B.B., Gaston K.J., Dean J., Barber E., Fuller R.A. (2015). Toward improved public health outcomes from urban nature. Am. J. Public Health.

[346] Lawton E., Brymer E., Clough P., Denovan A. (2017). The relationship between the physical activity environment, nature relatedness, and the psychological well-being benefits of regular exercisers. Front. Psychol..

[347] Nisbet E.K., Zelenski J.M., Murphy S.A. (2011). Happiness is in our nature: Exploring nature relatedness as a contributor to subjective well-being. J. Happiness Stud..

[348] Yeh H., Stone J., Churchill S., Brymer E., Davids K. (2017). Physical and emotional benefits of different exercise environments designed for treadmill running. Int. J. Environ. Res. Public Health.

[349] Yeh H., Stone J., Churchill S., Wheat J., Brymer E., Davids K. (2016). Physical, psychological and emotional benefits of green physical activity: An ecological dynamics perspective. Sports Med..

[350] Brymer E., Davids K., Mallabon L. (2014). Understanding the psycholgical health and well-being benefits of physical activity in nature: An ecological dynamics analysis. Ecopsychology.

